# Supplementary material for: An Optically Induced Dielectrophoresis (ODEP)-Based Microfluidic System for the Isolation of High-Purity CD45neg/EpCAMneg Cells from the Blood Samples of Cancer Patients—Demonstration and Initial Exploration of the Clinical Significance of These Cells
Source: Micromachines (Basel). 2018 Oct 31;9(11):563. doi: 10.3390/mi9110563 (PMC6266761; doi:10.3390/mi9110563)
Supplement: Supplementary file 1 [file micromachines-09-00563-s001.zip › micromachines-379917- supplymentary/CJL_supplementary information/CJL_Supplementary information_Micromachines.docx]

**Supplementary information**

**An Optically Induced Dielectrophoresis (ODEP)-Based Microfluidic System for the Isolation of High-Purity CD45^neg^/EpCAM^neg^ Cells from the Blood Samples of Cancer Patients—Demonstration and Initial Exploration of the Clinical Significance of These Cells**

Chia-Jung Liao ^1,†^, Chia-Hsun Hsieh ^2,†^, Tzu-Keng Chiu ^3^, Yu-Xian Zhu ^1^, Hung-Ming Wang ^2^, Feng-Chun Hung ^1^, Wen-Pin Chou ^1^ and Min-Hsien Wu ^1,2,4,^*


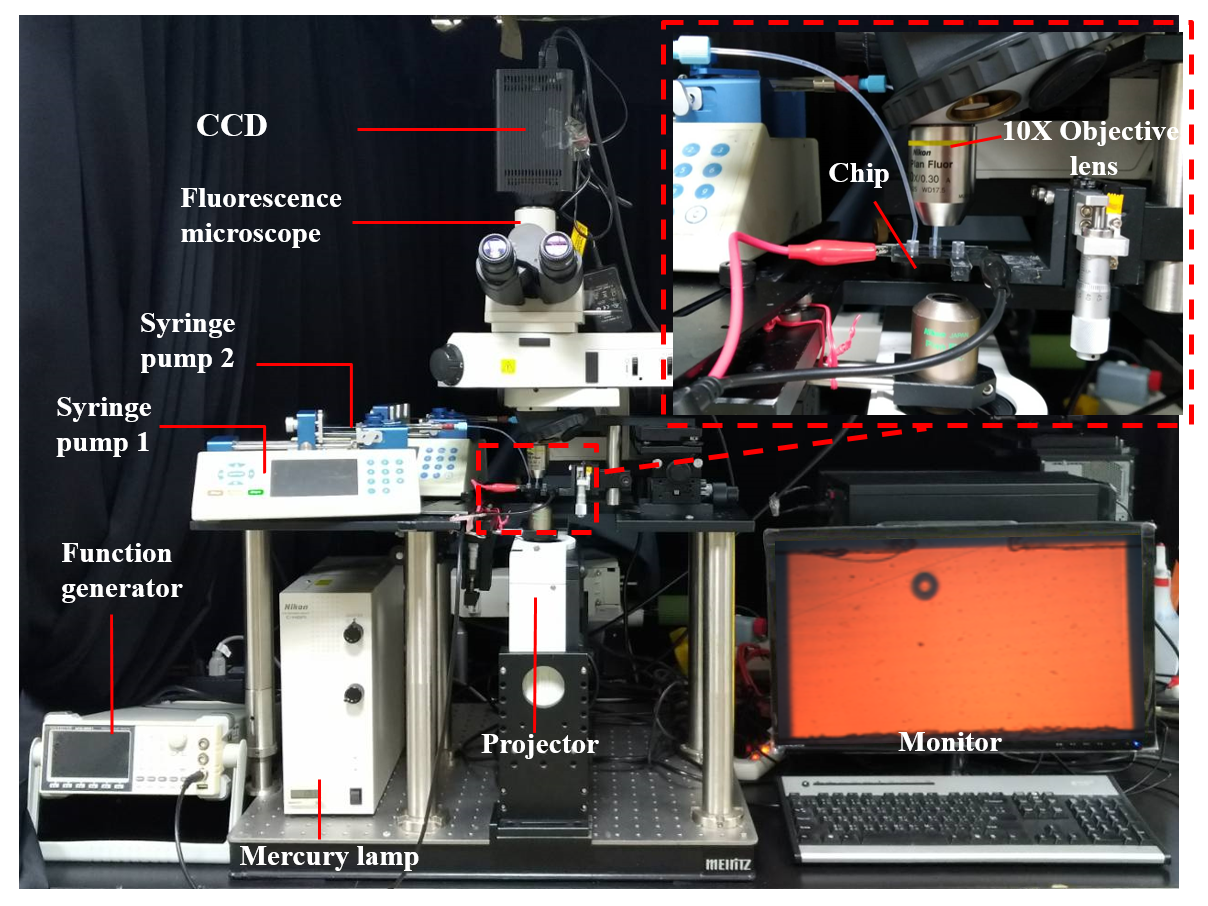


**Fig. S1** Photograph of the overall experimental setup.

| **Table S1** Gene expression status of CD45^neg^/EpCAM^neg^ nucleated cells isolated from the blood samples of healthy donors and head-and-neck cancer patients. | | | | | | | | | | | | | | | | |
| --- | --- | --- | --- | --- | --- | --- | --- | --- | --- | --- | --- | --- | --- | --- | --- | --- |
| Biological function | Gene name | Healthy donor | | | | |  | Head-and-neck cancer patient | | | | | | | | |
|  |  |  |  |  |  |  |  | Local | | | | |  | Metastatic | | |
|  |  | #1 | #2 | #3 | #4 | #5 |  | #1 | #2 | #3 | #4 | #5 |  | #1 | #2 | #3 |
| EMT-related | EpCAM | － | － | － | － | － |  | － | － | － | － | － |  | － | － | － |
|  | CK19 | － | － | － | － | － |  | － | － | － | － | － |  | － | － | ＋ |
|  | Vimentin | － | ＋ | － | － | － |  | ＋ | ＋ | － | ＋ | － |  | ＋ | ＋ | ＋ |
|  | SNAIL1 | － | － | － | － | － |  | － | － | － | － | － |  | － | － | ＋ |
| MDR-related | MRP1 | － | － | － | － | － |  | － | － | － | ＋ | － |  | － | ＋ | ＋ |
|  | MRP2 | － | － | － | － | － |  | － | － | － | － | － |  | － | － | － |
|  | MRP4 | － | － | － | － | － |  | － | － | － | － | － |  | － | － | － |
|  | MRP5 | － | － | － | － | － |  | ＋ | － | － | － | － |  | ＋ | － | － |
|  | MRP7 | － | － | － | － | － |  | － | － | － | － | － |  | － | － | － |
| CSC-related | NANOG | － | － | － | ＋ | － |  | － | － | － | － | ＋ |  | － | ＋ | － |
|  | OCT4 | － | ＋ | － | － | － |  | ＋ | － | － | － | ＋ |  | ＋ | ＋ | － |
